# A Real‐World Cross‐Sectional Study on Suspected Mast Cell Activation Syndrome: Multiple Heterogeneous Symptoms Identified

**DOI:** 10.1002/clt2.70184

**Published:** 2026-07-03

**Authors:** Annika Gutsche, Thomas Buttgereit, Rüdiger Buchkremer, Jens Panse, Nina Kreddig, Olga Ptock, Stephanie Roll, Weronika Grabowska, Felix Aulenbacher, Martin Metz, Polina Pyatilova, Pascale Salameh, Manuel P. Pereira, Frank Siebenhaar

**Affiliations:** ^1^ Charité – University Medical Center Berlin, Corporate Member of Freie Universität Berlin and Humboldt‐Universität zu Berlin Institute of Allergology Berlin Germany; ^2^ Fraunhofer Institute for Translational Medicine and Pharmacology ITMP, Immunology and Allergology Berlin Germany; ^3^ Institute of IT Management and Digitization Research (IFID) FOM University of Applied Sciences in Economics and Management Düsseldorf Düsseldorf Germany; ^4^ Department of Oncology, Hematology, Hemostaseology and Stem Cell Transplantation University Hospital RWTH Aachen Center for Integrated Oncology Aachen, Bonn Cologne, Duesseldorf (CIO ABCD) Aachen Germany; ^5^ Department of Medical Psychology and Medical Sociology Faculty of Medicine Ruhr University Bochum Bochum Germany; ^6^ Institute of Social Medicine, Epidemiology and Health Economics Charité‐Universitätsmedizin Berlin Berlin Germany; ^7^ Institute for Biostatistics and Informatics in Medicine and Ageing Research Rostock University Medical Center Rostock Germany; ^8^ School of Medicine Lebanese American University Beirut Lebanon; ^9^ Medical School University of Nicosia Nicosia Cyprus; ^10^ Institut National de Santé Publique, Epidémiologie Clinique et Toxicologie–Liban (INSPECT‐LB) Beirut Lebanon

**Keywords:** machine learning, MCAS, symptoms

## Abstract

**Background:**

A variety of clinical signs and symptoms have been described in patients suspected of having mast cell activation syndrome (MCAS), however, symptom patterns and linked organ systems were not yet described.

**Methods:**

This cross‐sectional online survey included adults with physician‐diagnosed self‐assessed or suspected MCAS between June and September 2021. Symptoms were collected as free‐text responses, and coded using the Medical Dictionary for Regulatory Activities (MedDRA). These data were then analyzed using an unsupervised machine learning approach to identify distinct participant clusters. Patient‐reported outcome measures (PROMs), including the PHQ‐2, GAD‐7, and SF‐36, were used to assess mental health symptoms and health‐related quality of life.

**Results:**

A total of 592 patients (mean age 45 years, 90% female) reported 400 symptoms in 58 categories. The hierarchical clustering algorithm failed to identify distinct patient clusters. We identified 256 (43%) patients with signs and symptoms aligning with the diagnostic criteria for MCAS including the most prevalent symptoms. Overall, patients infrequently reported mast cell‐mediated skin symptoms, such as pruritus (13%), wheals (9%), flushing (7%), or angioedema (1%). We identified patients with comorbid signs of depression (39%), general anxiety (34%), and impaired quality of life (28%).

**Conclusion:**

Patients with suspicion of MCAS exhibit highly heterogeneous symptoms, whereas almost 50% report symptoms corresponding to the first diagnostic criterion for MCAS. Because over a third of the patients were tested positive for other diseases, we recommend considering common and more treatable diagnoses when evaluating patients with suspected MCAS.

## 1 Introduction

Mast cell activation syndrome (MCAS) is an immunological condition caused by inappropriate and excessive release of proinflammatory mast cell (MC) mediators, resulting in the occurrence of related severe and episodic signs and symptoms [1, 2]. The condition is divided into primary MCAS (mastocytosis and monoclonal MCAS), secondary MCAS (known trigger factors, IgE‐dependent allergic reactions or underlying diseases), and idiopathic MCAS where no clear cause of mast cell activation (MCA) is evident [3, 4]. This study focused on the characterization of symptom patterns reported by patients with suspected MCAS.

Patients suspected of having MCAS commonly report a broad spectrum of symptoms, the underlying mechanisms of which often remain incompletely understood. Because of the frequently nonspecific nature of these symptoms, MCAS is increasingly considered as a diagnosis, whereas the true nature of the symptomatology often remains unidentified and might be caused by other diseases or factors. The criteria used to diagnose MCAS are still in discussion, with proposed criteria published by two independent groups [2, 5]. For the purposes of the current study, we relied on the Vienna Criteria, as agreed upon by the international ECNM‐AIM consensus group [2]. The Vienna Criteria propose three obligatory diagnostic criteria for determining MCAS. This paper focuses on the first criterion, as this is the main reason patients seeking medical attention: the presence of severe, recurrent, and episodic symptoms involving at least two different organ systems, including urticaria, redness, pruritus, angioedema, nasal congestion, nasal pruritus, wheezing, throat swelling, hoarseness, headache, hypotensive syncope, tachycardia, abdominal cramps, and diarrhea [2]. The other two criteria, increased serum tryptase level above baseline by 20% plus 2 ng/mL during an episode and symptom relief from treatment with MC‐stabilizing agents or drugs targeting MC mediators, are not considered further in this study, as the open survey did not allow for the verification of these criteria.

This study aimed to characterize all signs and symptoms reported by a sizable population of patients suspected of having MCAS. First, we sought to identify patients who reported typical signs and symptoms according to the first diagnostic criterion for MCAS (described above). Subsequently, we intended to classify patients into distinct groups based on their reported symptoms. Lastly, we screened patients for concomitant anxiety and depression that might be associated with their disease condition.

## 2 Methods

### 2.1 Study Population and Data Collection

In this cross‐sectional study, adults aged 18 years or older were included if they self‐reported suspected MCAS, whether they described it as physician‐diagnosed or self‐assessed; no clinical confirmation of MCAS was performed.

Ethical approval was obtained from the local ethics committee (EA1/274/20), and all patients provided online informed consent.

To facilitate patient recruitment, the advocacy group proactively announced and disseminated information about the study via patient networks. The URL link to the survey was published on the advocacy group website. The questionnaire consisted of 62 questions, 52 of them with pre‐defined responses and ten with free text, divided into five sections: demographics, diagnosis and disease history, health‐related quality of life, symptoms and disease condition at onset of disease, and current symptoms and disease condition. Further, the questionnaire integrated the Short Form 36 (SF‐36), the Generalized Anxiety Disorder 7 (GAD‐7), and the Patient Health Questionnaire‐2 (PHQ‐2). The SF‐36 is a widely used tool for measuring health‐related quality of life. It consists of 36 questions across eight subscales (PF, RP, BP, GH, VT, SF, RE, and MH), which are summarized into two component scores: physical (PCS) and mental health (MCS). The scoring ranges from 0 to 100, and 100 represents a perfect health‐related quality of life [6].

The GAD‐7 assesses key symptoms of generalized anxiety over the past 2 weeks. Scores range from 0 to 21, with > 5, > 10, and > 15 indicating mild, moderate, and severe anxiety levels, respectively [7]. The PHQ‐2 test is used to screen for the presence of depression, indicating depression when both questions are answered with “yes” [8].

### 2.2 Data Management

Patients were asked separately to name a) up to five episodic and recurrent symptoms and b) up to five permanent symptoms they experienced during the last 6 months in free text form. Referring to the Vienna Criteria, as previously described. Additionally, patients had the opportunity to name any other symptoms in a separate free text form. The free text data entry of current episodic and permanent symptoms was cleaned using a three‐step process. First, patient responses were extracted, followed by merging identical‐meaning terms. Subsequently, to ensure comparability in the analysis, all patient responses regarding symptoms (400 differently named symptoms) were standardized using the terminology provided by the Medical Dictionary for Regulatory Activities (MedDRA) [9]. And thirdly, if terms were ambiguous and could not be coded according to the MedDRA classification, they were categorized into a consented term by a multidisciplinary expert group (FS, JS, RB, OB, and AG). Finally, all MedDRA‐coded symptoms were aggregated to meaningful symptom categories and imputed into the cluster algorithm. After a thorough screening, discussion, and merging processes, a consensus was reached on 58 distinct symptom categories. Further, all patients were screened for symptoms consented in the diagnostic Vienna criteria of MCAS, that is, wheals, redness, pruritus, angioedema, nasal congestion, nasal pruritus, wheezing, throat swelling, hoarseness, headache, hypotensive syncope, tachycardia, abdominal cramps, and diarrhea. Additionally, following the first criterion, symptom constellations were examined to determine whether they originated from at least two distinct organ systems, defined by MedDRA's standard ontology for system organ classes (SOC) [9].

### 2.3 Statistical Analysis

We applied an unsupervised machine learning technique, to identify patterns and relationships in data without labeled training data to uncover the dataset's natural structure. Specifically, divisive hierarchical clustering was used to find clusters most similar within their group and as different as possible from other clusters [10, 11].

Proximity was assessed using a dissimilarity matrix calculated by the “Gower” distance, which was suited for categorical variables [12]. An “average linkage” was provided as a linkage method, which calculates the distance to another cluster's point over all a cluster's points and averages these distances. This approach results in observations being close within a cluster and far away from observations of other clusters. Before fitting the clustering algorithm, all variables were transformed from text to numeric into a matrix using a standard scale of zeros (absence of symptoms) and ones (presence of symptoms) [13]. Only symptoms recorded by at least 5% of the study population were included in the algorithm to reduce high dimensionality. A dendrogram visually depicted the cluster hierarchy, and the elbow method was used to determine the optimal number of clusters in the data. This method involves plotting the sum of squared distances from each point to its assigned cluster center for different numbers of clusters [12].

Based on standard scoring guidelines, patients were categorized according to specific cut‐offs corresponding to the questionnaires used in the study: Those likely to have depression, answering both question with “yes” resulting in PHQ‐2 positive, showing low mental health‐related quality of life (SF‐36 MCS < 30), and experiencing higher anxiety levels (GAD‐7 ≥ 10). Symptoms and related organ classes were descriptively analyzed for all those patients who suffered from depression, low mental health, or higher levels of anxiety. All statistical analyses were performed using RStudio 2022.12.0.

## 3 Results

### 3.1 MCAS Is Primarily Suspected in Middle‐Aged Females With a Long Disease Duration

In 592 patients with suspected MCAS (*N* = 1187, N included = 934, and N analyzed = 592), the mean (± standard deviation) age was 45 ± 13 years, and 90% were female, 9% were male, and 1% identified as diverse. The mean age of females and males were similar (female: 45 ± 13 years [range 19–80] and male: 44 ± 12 years [range 24–69]). Most patients lived in Germany, followed by Austria, Switzerland, and other countries (91%, 5%, 3%, and 1%, respectively, Table 1). One in two patients (51%) experienced symptoms for longer than 10 years, and 20% between 4 and 7 years, whereas 2% had symptoms for less than 1 year. From free text data entry, we identified 400 different symptoms reported by the patient cohort.

**TABLE 1:** Demographics of the total study population (*N* = 592).

	Total study population (*N* = 592)
Age	
mean (SD)	45 (13)
median	45
Gender	
female	533 (90%)
male	54 (9%)
diverse	5 (1%)
Country of residency	
Germany	536 (91%)
Austria	27 (5%)
Switzerland	20 (3%)
other	9 (1%)
Employment status	
employed	294 (50%)
not employed	286 (48%)
not applicable	12 (2%)
Symptoms duration	
< 1 year	14 (2%)
1–3 years	78 (13%)
4–7 years	118 (20%)
8–10 years	80 (14%)
> 10 years	302 (51%)
Disease duration	
< 1 year	26 (4%)
1–3 years	119 (20%)
4–7 years	163 (28%)
8–10 years	66 (11%)
> 10 years	218 (37%)

Abbreviation: SD, standard deviation.

### 3.2 Patients With Suspected MCAS Report Heterogeneous Signs and Symptoms

**FIGURE 1:**
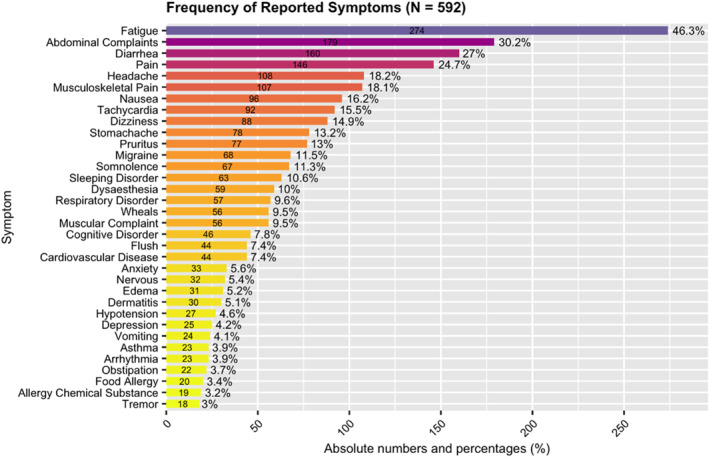
Frequency (%) of symptoms reported by the study population (*N* = 592). This bar chart illustrates the distribution of various symptoms reported by the study population (*N* = 592). Only symptoms that were reported by at least 3% of patients were included in this plot.

Following the first diagnostic criterion of the Vienna Criteria, episodic symptoms are of particular importance in diagnosing MCAS. In total, patients reported 58 different signs and symptoms that occurred episodically during the previous six months. On average, patients reported four episodically occurring symptoms (SD 1.5, range 1–6) that affected any three different organ systems (SD 1.2, range 1–6). The most frequently reported signs and symptoms were fatigue (46%), abdominal complaints (30%), diarrhea (27%), and unspecified pain (25%) (Figure 1). Fatigue and unspecified pain do not correspond to the first criterion of MCAS and are attributed to general disorders (Table 2). According to the MedDRA, general disorders encompass a broad range of conditions that do not fit neatly into other categories and impact overall body function or general well‐being such as fatigue, fever, unspecified pain, and malaise. Consistent with this, most patients (94%) also reported permanently present symptoms, with 49 different signs and symptoms documented. The most frequently reported permanent symptoms were fatigue (58.7%), unspecified pain (28.4%), abdominal complaints (26.1%), musculoskeletal pain (18.8%), and sleep disorders (15.2%). MC‐mediated skin symptoms, such as pruritus, wheals, flushing, and angioedema, were reported at 13%, 9%, 7%, and 1%, respectively.

**TABLE 2:** Frequencies (%) of top 5 reported symptoms by the entire study population (*N* = 592).

SOC^a^	Symptom	Symptom frequency (%)
General disorders	Fatigue	274 (46%)
	Pain	146 (25%)
Gastrointestinal disorders	Abdominal complaints	179 (30%)
	Diarrhea	160 (27%)
Nervous system disorders	Headache	108 (18%)

^a^ System organ class (SOC).

### 3.3 A Minor Portion of Patients Reported Signs and Symptoms Corresponding to the Diagnostic Criteria for MCAS

We identified 23/592 patients (4%) who exclusively reported signs and symptoms of at least two episodic symptoms from two different organ classes that correspond to the first diagnostic criterion of MCAS (urticaria, redness, pruritus, angioedema, nasal congestion, nasal pruritus, wheezing, throat swelling, hoarseness, headache, hypotensive syncope, tachycardia, abdominal cramps, and diarrhea) and no other additional symptoms. About 43% (256/592) of patients reported at least two episodic symptoms from two different organ classes as defined in the first criterion for MCAS but also reported on other additional symptoms.

**FIGURE 2:**
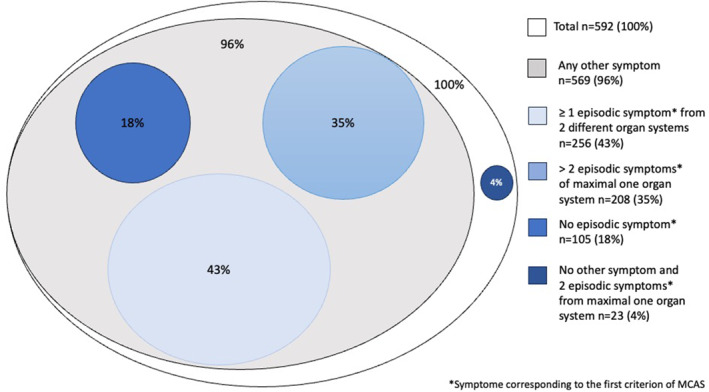
Percentage distribution of signs and symptoms. This figure shows the percentage distribution of patients who correspond to the signs and symptoms of the first diagnostic criterion for MCAS.

Of the remaining 313 patients (53%), 105/569 patients (18%) did not report any of the symptoms defined in the first diagnostic criterion for MCAS, whereas 208/569 patients (35%) reported one or more episodic symptoms from only one organ system (Figure 2).

### 3.4 Patients Who Meet the First Diagnostic Criterion for MCAS Report on Average Three Symptoms Unrelated to the Diagnostic Criteria

On average, patients who met the first criterion for MCAS (*n* = 256, 43%) reported a total of 5 (SD 1.2, range 3–9) symptoms while also reporting 3 (SD 1.3, range 1–7) symptoms which were unrelated to the first criterion for MCAS. The most frequently reported episodic symptoms among those patients who met the first criterion for MCAS were diarrhea (47%) and abdominal complaints in 42%. General disorders, such as fatigue, were present in 43% of patients, whereas unspecified pain was reported by 25%. Nervous system disorders included headaches in 29% and dizziness in 25% of patients. Cardiac symptoms, specifically tachycardia, were noted in 26% of cases. Skin symptoms related to mast cells were reported less frequently. Pruritus and wheals were noted by 18% and 17% of patients, whereas flush and angioedema were reported by 14% and 2%, respectively (Table 3). Among patients (*n* = 23,4%) who exclusively reported episodic symptoms, gastrointestinal issues were common, with 30% experiencing abdominal complaints and 17% reporting diarrhea. Pruritus and urticaria (wheals) were reported in 26% of those patients, corresponding to 6 individuals. Angioedema and flushing were not documented within the 4% (Table 3).

**TABLE 3:** Frequencies (%) of episodic symptoms of patients who report signs and symptoms corresponding to the diagnostic criteria for MCAS.

SOC	Symptom	Frequency (%) of all patients with episodic symptoms^a^ (*N* = 256)	Frequency (%) of patients who exclusively report episodic symptoms^a^ (*N* = 23)
Gastrointestinal disorders	Diarrhea	121 (47%)	4 (17%)
	Abdominal complaints	119 (42%)	7 (30%)
General disorders	Fatigue	112 (43%)	—
	Pain (unspecified)	63 (25%)	—
Nervous system disorders	Headache	74 (29%)	2 (9%)
	Dizziness	65 (25%)	1 (4%)
Cardiac disorders	Tachycardia	67 (26%)	1 (4%)
Skin and subcutaneous tissue disorders	Pruritus	47 (18%)	6 (26%)
	Urticaria (wheals)	43 (17%)	6 (26%)
	Flush	35 (14%)	
	Angioedema	5 (2%)	

^a^ Episodic symptoms corresponding to the diagnostic criteria for MCAS.

### 3.5 An Unsupervised Machine Learning Approach Failed to Identify Distinct Symptom Patterns

**FIGURE 3:**
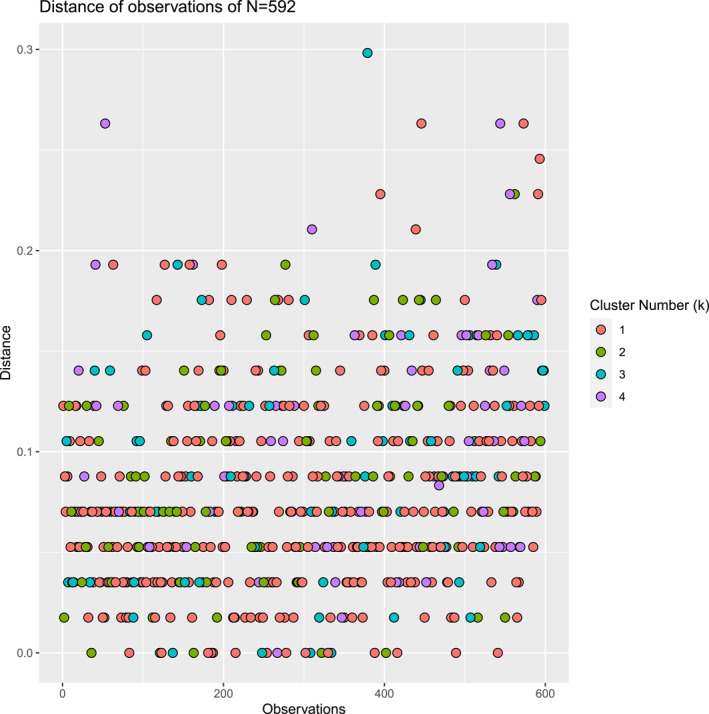
Hierarchical cluster analysis on symptom data. The figure depicts the distances of 592 observations *related* to the optimal number of identified clusters, which is four (*k* = 4). Each observation is represented by a dot, with the position on the *x*‐axis indicating the observation's index and the position on the *y*‐axis showing its distance from the clustering center. No coherent clusters were observed.

The application of an unsupervised machine learning technique failed to detect distinct patterns of symptoms that would lead to forming separate clusters Figure 3. Specifically, machine learning‐based “Gower” clustering with linkage and use of the elbow method of dimensionally reduced data did not detect pronounced inflection points in the elbow plot and the calculated distances between the optimal number of clusters (*k* = 4), as illustrated in Supporting Information S1: Figure S2. The application of the algorithm to ascertain the optimal number of clusters has been executed; however, the visualization in Figure 3 indicates that the data does not segregate into meaningful clusters. Thus, although underlying diseases and general symptoms do not necessarily preclude MCA, symptoms may be attributed to other underlying diseases.

### 3.6 Patients Reported a High Frequency of Symptoms Associated With Depression and Anxiety

Half of the participating patients (301/592, 51%) screened positive on at least one of the selected screening instruments (PHQ‐2: two “yes” responses; GAD‐7: ≥ 10; SF‐36 MCS: < 30), whereas 18% (108/592) screened positive on all three tests.

Among the 592 participants, 230 (39%) screened positive on the PHQ‐2 (both items endorsed), 198 (33.5%) on the GAD‐7 (score ≥ 10), and 167 (28.2%) on the SF‐36 mental component summary (MCS; score < 30). On the contrary, Figure 1 shows that 4.2% of participants self‐reported depression and 6% noted anxiety among their symptoms.

When screening results were compared with self‐reported assessments, 39% of participants screened positive on the PHQ‐2, whereas only 4.2% (25/592) self‐reported depression. GAD‐7 ≥ 10 was met by 198/592 (33.4%), whereas self‐reported anxiety was 5.6% (33/592). SF‐36 MCS < 30 was observed in 167/592 (28.2%); no corresponding self‐report item was collected. Despite the lower self‐reported rates of depression (4.2%) and anxiety (5.6%), the standardized screening instruments indicate higher prevalence.

## 4 Discussion

This is the first study to comprehensively evaluate the signs and symptoms reported by a large population of patients suspected of having MCAS in Germany, Austria, and Switzerland by collecting free‐text data and standardizing them through MedDRA coding [9]. In addition to the recording of symptoms, the study compared the results of symptom patterns to the results of validated screening instruments for quality‐of‐life impairment, depression, and anxiety. Our findings demonstrate that attempting to identify common and disease‐defining symptom patterns based on patient‐reported signs and symptoms using an unsupervised cluster algorithm failed. In other words, symptoms reported by patients with suspected MCAS remain heterogeneous and diverse. Interestingly, patients who reported symptoms aligning with the diagnostic criteria for MCAS (e.g., urticaria, flushing, pruritus, angioedema, nasal congestion, nasal pruritus, wheezing, throat swelling, hoarseness, headache, hypotensive syncope, tachycardia, abdominal cramping, and diarrhea) did not show a distinct overall symptom pattern compared to the entire study population, as they also reported a high number of symptoms unrelated to what is described as typical for MCAS by the Vienna criteria [2]. Debate persists about how MCAS should be defined and diagnosed [14, 15, 16]. At the same time, increased awareness has led to various manifestations being more frequently attributed to MCAS.

Consequently, the cohort investigated in this study likely includes individuals who do not meet the Vienna criteria for MCAS. This potential misclassification is a plausible cause of the observed discrepancy between patient reported symptoms and the proposed diagnostic criteria for MCAS. Alternatively, the proposed criteria may be too restrictive to fully capture MCAS in its plethora of symptomatology, as proposed by some experts, should it indeed be as heterogenous as the data suggest. It remains to be determined whether the symptoms proposed as typical by the Vienna criteria can be considered disease‐defining and serve as a common symptom combination that characterizes MCAS as a distinct disease entity.

Nevertheless, many symptoms reported by patients with suspected MCAS, such as dizziness, are rather nonspecific. Although they may be associated with MCAS, they cannot be considered disease‐determining in and of themselves, as they can also be caused by numerous other conditions unrelated to MCA [17, 18]. We found that clearly defined MCA‐related skin symptoms are rather rare. Less than 15% of patients reported itching, and less than 10% of patients reported urticaria, flushing, and angioedema. Among the patients (*n* = 256) who reported signs and symptoms consistent with the first criterion for MCAS, less than 20% exhibited mast cell‐mediated skin symptoms. Even in the 4% of patients who exclusively reported on signs and symptoms of the first diagnostic criterion for MCAS, mast cell‐mediated skin symptoms were only reported in 26% of cases. Consequently, true MCAS is either underrepresented in the current sample, or those symptoms are less typical than previously thought.

A more rigorous examination is needed to determine whether symptoms meet the proposed disease‐specific criteria to confirm or exclude the diagnosis, including comorbid conditions. Mihele et al. state that “there is still no certainty as to whether cases of MCAS are not missed versus the possibility of overdiagnosis” [3]. Certain symptoms may correlate with other medical conditions [17].

Previous studies have shown a high prevalence of psychological complaints such as anxiety and depression in MCAD, predominantly in mastocytosis [18], but also MCAS [19]. These complaints can be primary, secondary or unrelated to MCA. In the cohort (*N* = 592), 18% (108/592) screened positive on all three instruments. Individually, 39% were PHQ‐2 positive, 33% met GAD‐7 ≥ 10, and 28% had SF‐36 MCS < 30, whereas only 4.2% and 5.6% self‐reported depression and anxiety, respectively. The gap between screening results and self‐reports likely reflects several factors: Firstly, validated screening tools may capture recent symptom severity and might be more sensitive than single self‐report items; secondly respondents may avoid labeling themselves as ‘depressed’ or ‘anxious’ because of concerns about stigma, a lack of formal diagnosis or recall bias; thirdly MCAS‐related somatic symptoms may overlap with depressive/anxiety items and can elevate scores [20]. Therefore, careful screening can ensure that patients receive timely and appropriate care, as previous studies have suggested an elevated risk of mental health issues in patient with MCAS.

Our study has several strengths and limitations. As for the former, our approach of using an open survey with free text items allowed us to collect a broad spectrum of clinical signs and symptoms of patients suspected of having MCAS and reaching a large sample size. To identify and remove potential duplicate responses, we implemented CAPTCHA verification and a manual review process. This study is limited by selection bias and reduced representativeness inherent to open, online, patient‐reported recruitment, with a marked demographic skew (predominantly female and Germany‐based participants). Moreover, as diagnoses were self‐reported and not clinically verified, we could not determine the presence of coexisting conditions (e.g., systemic mastocytosis, confirmed IgE‐mediated food allergy, or prior allergic anaphylaxis) or stratify participants accordingly. Therefore, the findings should be interpreted cautiously and may not generalize to clinically confirmed MCAS populations, particularly those managed in specialist care. Finally, we acknowledge the potential for recall bias and information bias, as participants were asked to report on events that may have occurred more than 5 years earlier. In addition, responses were self‐reported and may have been influenced by social desirability bias. A further limitation is that, although symptom overlap across organ systems could be assessed, the survey did not capture sufficiently detailed episode‐level data to evaluate whether recurrent multi‐system complaints occurred in a stereotypic pattern, which is clinically relevant for the assessment of suspected MCAS. Complicating MCAS research, the diagnostic criteria for MCAS are not yet finalized. There are two prominent sets of proposed criteria, respectively criticized for being too restrictive or too inclusive. We chose to implement the more restrictive Vienna criteria in this study. Given the marked symptom heterogeneity and high screening positivity for anxiety and depression, it is possible that a proportion of participants had functional somatic/psychosomatic disorders either as a primary condition or comorbidity; however, this could not be confirmed or excluded in this self‐reported survey without clinical diagnostic evaluation. This further supports the need for multidisciplinary assessment and differential diagnosis.

In conclusion, patients with suspicion of MCAS exhibit highly heterogeneous symptoms, but only a few of them include well‐defined MC‐mediated symptoms. Despite our attempts to cluster the reported symptoms, a distinct population did not emerge. When evaluating patients with suspected MCAS, we recommend to also considering common and better‐defined differential diagnoses and potential comorbidities with similar symptomatology before deciding whether further suspicion of MCAS is justified or a MCAS diagnosis is finally established. Anxiety, depression, and low mental health are frequent in the current sample. Apart from related psychiatric symptoms, no other symptoms differed significantly between patients with or without psychological complaints. Future evaluations should carefully review all plausible differential diagnoses within practical constraints, beyond anxiety and depression. We lack a clear understanding of the effector of MCAS, and a distinct set of symptoms related to MCA. To provide conclusive evidence of the underlying pathology, the first step requires thorough studies and empirical evidence that go beyond the mere characterization and definition of the disease.

## Author Contributions

Annika Gutsche was involved in study planning, including study design, protocol writing, ethical approval, data management, and the statistical analysis plan, as well as survey development and data collection. She managed data export, statistical analysis, and manuscript writing. Frank Siebenhaar and Rüdiger Buchkremer supervised the study and were involved in all processes. Rüdiger Buchkremer initiated the study together with MCAS Hope e. V., a non‐profit support association based in Germany that focuses on the needs of individuals living with Mast Cell Activation Syndrome. Stephanie Roll and Weronika Grabowska were involved in study planning, survey development, writing the statistical analysis plan, and proofreading the manuscript. Pascale Salameh provided statistical consultation. The consortium, including Nina Kreddig, Olga Ptock, and Jens Panse, was involved in study planning, patient recruitment, survey development, and proofreading the manuscript. Felix Aulenbacher supported the transformation of text into numeric data. Martin Metz, Thomas Buttgereit, Pascale Salameh, Manuel P. Pereira, Polina Pyatilova, and Martin Metz proofread the manuscript.

## Funding

The authors have nothing to report.

## Conflicts of Interest

A. Gutsche has no conflicts of interest concerning this work. T. Buttgereit is or recently was a speaker and/or advisor for and/or has received research funding from Aequestive, CSL Behring, BioCryst, KalVista, Pharming, Pharvaris, Roche, Medac, GSK, Sanofi‐Aventis, Swixx BioPharma, Takeda/Shire and Novartis. R. Buchkremer has no conflicts of interest concerning this work. J. Panse Panse is or recently was a speaker and/or advisor for and/or has received research funding (to his institution from Alexion, Amgen, Apellis, Astra Zeneca, Blueprint Medicines, Boehringer Ingelheim, BMS Cogent, MSD, Omeros, Roche, Samsung Bioepis, Sandoz, Sanofi, SOBI. N. Kreddig leads the enterprise Mastzellenhilfe and serves on the governing board of MCAS Hope e.V. O. Ptock has no conflicts of interest concerning this work. S. Roll has no conflicts of interest concerning this work. W. Grabowska has no conflicts of interest concerning this work. F. Aulenbacher has no conflicts of interest concerning this work. M. Metz is or recently was a speaker and/or advisor for AbbVie, Allmiral, ALK‐Abello, Amgen, AstraZeneca, argenx, Bayer, Beiersdorf, Celldex, Celltrion, Escient, Galderma, gsk, Incyte, Jasper, Novartis, Pharvaris, Pfizer, Regeneron, Sanofi, Teva, ThirdHarmonicBio, Vifor P. Pyatilova has no conflicts of interest to declare concerning this work, P. Salameh has no conflicts of interest to declare in relation to this work. M. P. Pereira MPP is an investigator for Allakos, Amgen, Celldex Therapeutics, Escient, Incyte, Mitsubishi, Sanofi and Trevi Therapeutics; and is an advisor or has received speaker honoraria/travel fees from AbbVie, Beiersdorf, Celltrion, CSL Behring, Doctorflix, Eli Lilly, Falk Foundation, FomF GmbH, GA2LEN, Galderma, Novartis, Pfizer, P.G. Unna Academy, Sanofi, StreamedUp, TouchMDT, Zai Lab. F. Siebenhaar is or recently was a speaker and/or advisor for and/or has received research funding from Allakos, Blueprint, Celldex, Cogent, Escient, Granular, GSK, Invea, Moxie, Noucor, Novartis, Sanofi/Regeneron, ThirdHarmonicBio, and UCB.

## Supporting information


Supporting Information S1

## Data Availability

The data that support the findings of this study are available from the corresponding author upon reasonable request.
