# Supplementary material for: A Real‐World Cross‐Sectional Study on Suspected Mast Cell Activation Syndrome: Multiple Heterogeneous Symptoms Identified
Source: Clin Transl Allergy. 2026 Jul 3;16(7):e70184. doi: 10.1002/clt2.70184 (PMC13331725; doi:10.1002/clt2.70184)
Supplement: Supplementary file 1 — Supporting Information S1 [file CLT2-16-e70184-s001.docx]

**Supplemental material**

**Supplemental Figure 1** Flowchart of study inclusion


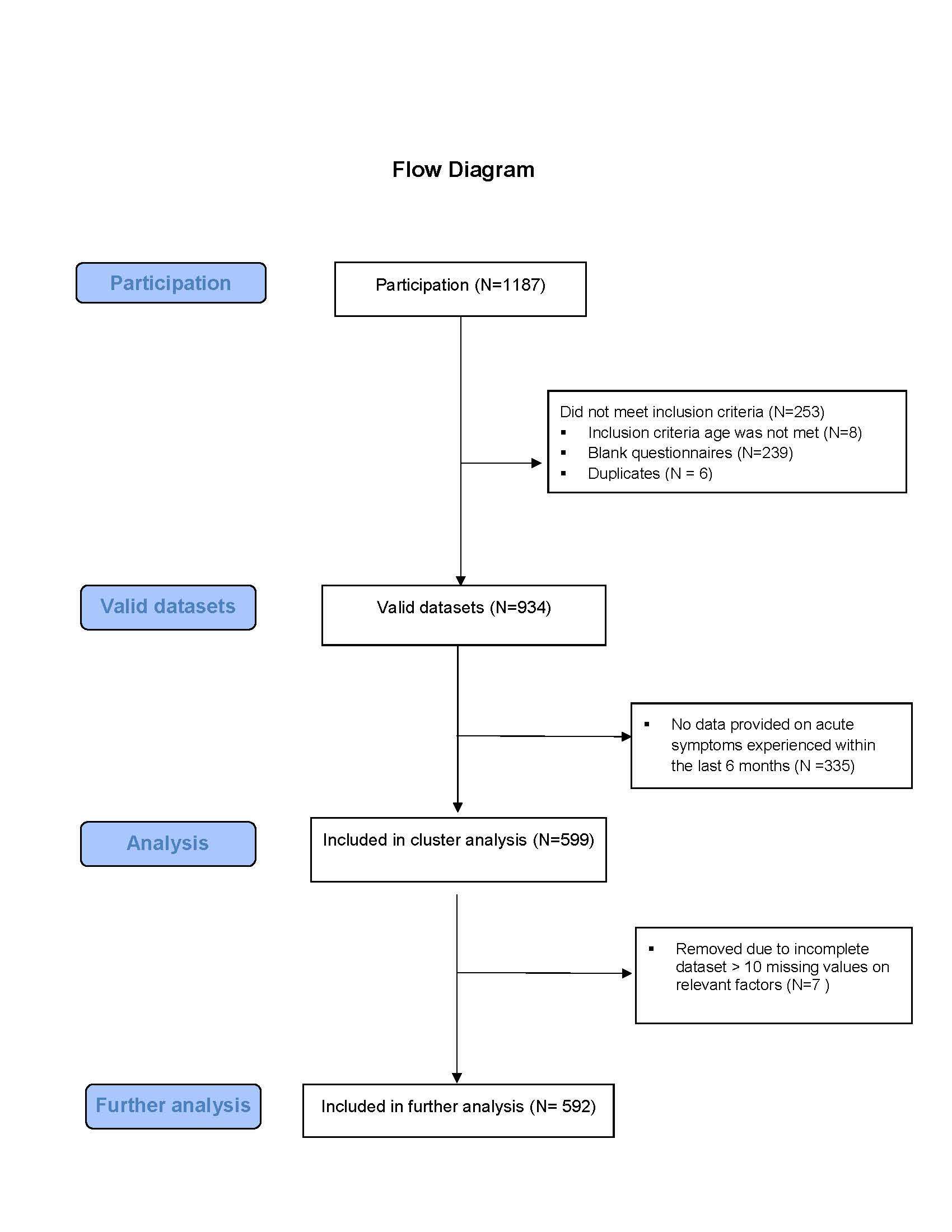


The flowchart illustrates the selection process for the study population, starting with 1187 participants. Out of these, 253 were excluded due to unmet inclusion criteria (8 for age, 239 for blank questionnaires, and 6 duplicates), resulting in 934 valid datasets. From these, 335 were excluded for not providing data on acute symptoms experienced within the last six months, leaving 599 for cluster analysis. An additional 7 datasets were excluded for having over 10 missing values on relevant factors, resulting in 592 datasets included in the further analyses.

**Supplemental Figure 2**: Elbow plot derived from hierarchical cluster analysis


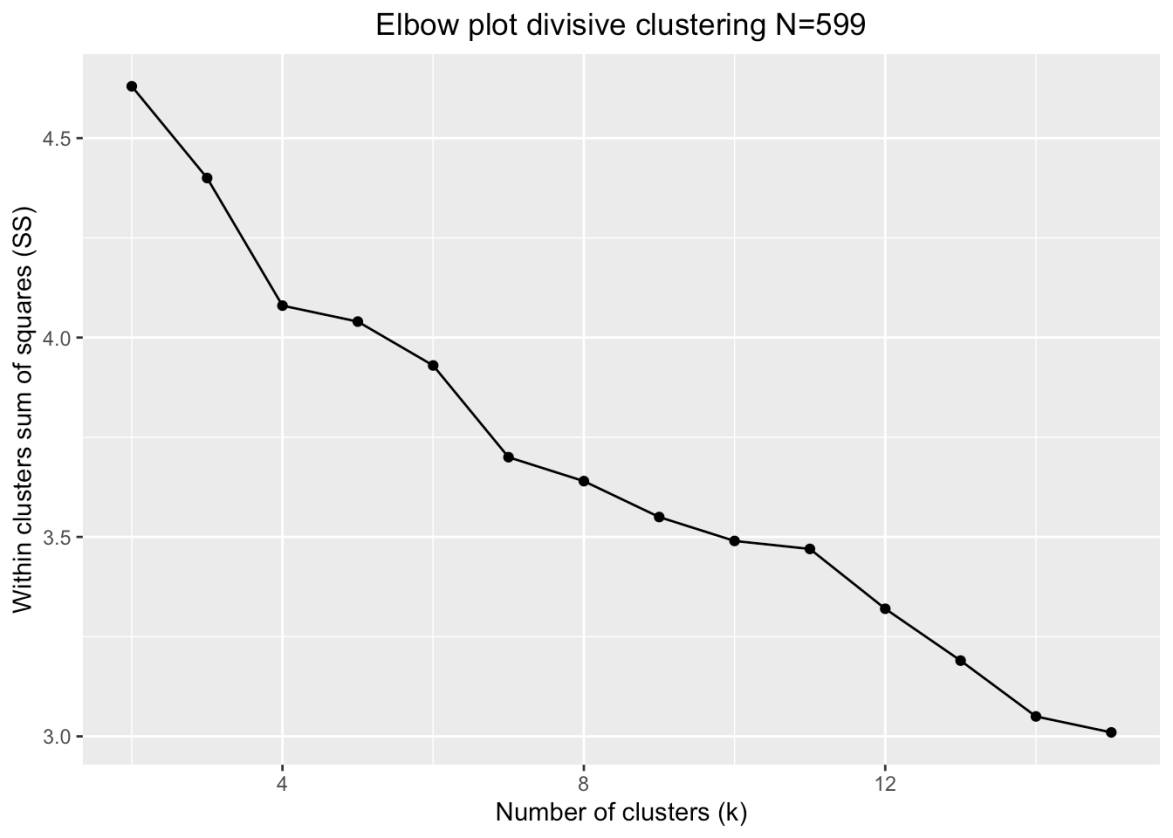


The 'elbow method' applied suggests that the optimal k (number of clusters) is 4, at the point just before the Within-Cluster SS starts to decline more slowly, representing the sum of the distances between the records and their respective cluster centers for each cluster. Lower values indicate tighter clustering of data points.
